# Empowered patients and informal care-givers as partners?—a survey study of healthcare professionals’ perceptions

**DOI:** 10.1186/s12913-023-09386-8

**Published:** 2023-04-26

**Authors:** Therese Scott Duncan, Sara Riggare, Ami Bylund, Maria Hägglund, Terese Stenfors, Lena Sharp, Sabine Koch

**Affiliations:** 1 Karolinska Institutet, LIME, Stockholm, S-171 77 Sweden; 2 Department of Women’s and Children’s Health, Uppsala University, Uppsala, Sweden; 3 Sophiahemmet University, Stockholm, Sweden; 4 Regional Cancer Centre Stockholm – Gotland, Stockholm Region, Sweden; 5 Department of Nursing, Umeå University, Umeå, Sweden

**Keywords:** Patient empowerment, Patient-centered care, Primary Health Care, Specialized Healthcare, Secondary care, Healthcare professionals’ perceptions, Sweden

## Abstract

**Background:**

More knowledge is needed regarding the perceptions of healthcare professionals when encountering empowered patients and informal caregivers in clinical settings. This study aimed to investigate healthcare professionals’ attitudes towards and experiences of working with empowered patients and informal caregivers, and perception of workplace support in these situations.

**Methods:**

A multi-centre web survey was conducted using a non-probability sampling of both primary and specialized healthcare professionals across Sweden. A total of 279 healthcare professionals completed the survey. Data was analysed using descriptive statistics and Thematic analysis.

**Results:**

Most respondents perceived empowered patients and informal caregivers as positive and had to some extent experience of learning new knowledge and skills from them. However, few respondents stated that these experiences were regularly followed-up at their workplace. Potentially negative consequences such as increased inequality and additional workload were, however, mentioned. Patients’ engagement in the development of clinical workplaces was seen as positive by the respondents, but few had own experience of such engagement and considered it difficult to be achieved .

**Conclusion:**

Overall positive attitudes of healthcare professionals are a fundamental prerequisite to the transition of the healthcare system recognizing empowered patients and informal caregivers as partners.

**Supplementary Information:**

The online version contains supplementary material available at 10.1186/s12913-023-09386-8.

## Introduction

The healthcare system has a focus on patient empowerment today with the goal to empower patients to become active participants in their care [1]. This requires not only an understanding of patients’ experiences from disease and illness, but also expectations of healthcare professionals providing patient involvement and organizational support for these efforts [2]. Empowered patients are described through several concepts in the literature, often as active, engaged, empowered, and knowledgeable participants, even though there is no consensus of the definition in the literature [3]. In the early 2000s the term *e-patient* was initially coined by Ferguson and Frydman inspired by the digital development within society which was reflected in many patients’ and informal caregivers’ behaviors [4]. E-patients were originally described as patients or informal caregivers (such as a family member or other persons with a close relationship to the patient) who used the Internet to find guidance regarding their own, or someone else’s, health challenge. E-patients use their own health data to learn from and engage in innovations of digital tools to meet health-related needs. They find ways to actively make their current situation more sustainable, rather than passively waiting for potential future solutions or cures [5–7]. A concept in close relation to e-patients, is *patient lead user* (“Spetspatienter” in Swedish) defined by Riggare [8] as:

“Patients or family members who take a larger responsibility for their own health and well-being. They meet their health-related challenges in a constructive and knowledge-based way, while taking their physical and mental abilities as well as capacity into account. Patient lead users make use of their own experiences to improve healthcare, on all levels of the system, for the sake of both themselves and other patients. Often you do not become a patient lead user by choice, it is something that you do to be able to manage and navigate the complex healthcare system” [8].

The concept of patient lead user originates from Von Hippel’s *lead users*, describing lay persons having a great need within a specific context, without any available solutions at the market. Lead users will then innovate solutions for that need, and the general market will follow [9, 10]. These concepts describe patients’ and informal caregivers’ active engagement, however their self-management tasks and healthcare system involvement are further described in a recent study of empowering behaviors of patients and informal caregivers. Here exploratory and influencing activities that patients and informal caregivers perform are characterized [11]. The exploratory empowering behaviors are labelled as *the self-care expert, the knowledge seeker, the academic, the patient researcher, the tracker, the coping-expert*, and *the exposed* and the influencing behaviors as *the innovator, the entrepreneur, the communicator, the mentor, the healthcare coordinator, the healthcare partner*, and *the activist* [11]. These empowering behaviors have evolved in parallel to the digital transformation in society [7] and require patients and informal caregivers to be involved and receive feedback. Due to the development of digital solutions for self-management, technological information systems, and focus on patient involvement during the 21st century in Sweden [1], patients’ and informal caregivers’ behaviors have changed. Swedish healthcare is nationally regulated, tax-funded, and locally administrated. Inhabitants of all social groups are entitled to a strong safety net since everyone have the same benefits and a maximum out-of-pocket cost [12, 13]. There is a high use of the Internet in the Swedish population (94% on a daily basis in 2022) [14], and, in line with other Scandinavian countries, long-term experiences of end user involvement in digital technology [15]. This might increase a movement towards more empowered patients and informal caregivers in Sweden.

However, studies researching healthcare professionals’ knowledge and perception of these patient behaviors are scarce. Some studies describe healthcare professionals’ concerns about patients’ online information seeking which might lead to stressful encounters with unrealistic expectations from patients [16, 17], as well as the concern of patients or informal caregivers finding harmful information [17]. Other studies show that healthcare professionals believe ideas and questions raised from patients’ online information searches could improve diagnostic decision making [18] and the patient-professional relationship [19]. There seem to be opportunities for healthcare professionals to ensure quality-controlled information access of their patients, for example by providing recommendations of websites, referrals to different peer-communities (e.g. patient associations), explaining or discussing research results with patients, or including patients’ own self-generated data into medical decision making [20]. Still, research indicate a continued concern from healthcare professionals regarding patients and informal caregivers searching medical information online [17, 20].

More knowledge is needed regarding the experiences and attitudes of healthcare professionals when encountering empowered patients and informal caregivers in clinical settings, and to what extent clinical workplaces offer strategies and support for this. Could empowered patients and informal caregivers contribute to improving the quality of health care? Therefore, the aim of this study was to investigate healthcare professionals’ (1) attitudes towards and (2) experiences of working with empowered patients and informal caregivers, and (3) perception of workplace support with regards to collaborating with empowered patient and informal caregivers.

## Method

### Study and survey design

This cross-sectional study had a descriptive design [21] with a multi-centre web survey conducted in Swedish. Using descriptions of empowering behaviors from a previous study as a guide [11], a study specific questionnaire was developed by the research team. The survey questions are presented in an additional file [see Additional file 1]. Before distributing the web survey, six cognitive interviews were performed to validate the questions. The web survey was pilot tested among 30 healthcare professionals (being part of the respondents) before the final distribution. The final web-survey consisted of a demographic section with five closed multiple-choice options, nine sections containing a total of 42 statements (5-point Likert-scale from *strongly disagree* to *strongly agree*) and nine free text options, as well as an additional section with four open-ended response questions. The open-ended questions and free text options were used as follow-ups to the closed multiple-choice options and non-mandatory. All other questions were mandatory to answer.

### Data collection

Non-probability sampling was performed, acknowledging the consequence of an exploratory sample not reaching the whole study population [22]. The link to the survey was first distributed to operational managers at different specialist healthcare settings managing oncology, diabetes, neurology, psychiatry, and rheumatic patients. These settings were representing the five patient driven innovations facilitating co-care within the research programme entitled “The patient in the driver seat”, that this study is part of. Managers distributed the link to their employees’ e-mail addresses. Two reminders were sent out. However, this approach resulted in a low response rate (18%). As a second distribution, an advertisement strategically targeting healthcare professionals with interest in health care, speaking Swedish, and age over 18 years old, was posted in social media (Facebook) for two weeks. The advertisement was distributed by Karolinska Institute’s Communications and public relations office, and reaching healthcare professionals within specialized, primary, and secondary healthcare settings in Sweden. Inclusion criteria for both samples were being a healthcare professional, older than 18 years, and Swedish speaking. The web survey was developed, distributed, and collected through Microsoft Teams, and data were collected between April 2021 and February 2022.

### Data analysis

A descriptive quantitative approach was used to analyse demographic characteristics and survey responses from a frequency perspective [21]. Similar responses were dichotomized according to being positive (“agree” and “strongly agree”) or being negative (“somewhat disagree” and “strongly disagree”). The analysis was performed using Microsoft Excel © [23]. Survey responses were summarised into three main topics based on the nine sections in the survey. The nine sections are displayed in an additional file [see Additional file 1]. A brief Thematic analysis was performed of the qualitative data from the open-ended responses, to see if the narrative changed. An inductive coding was performed when grouping codes generated from the data into categories. From this a deductive approach was performed using the three main topics to locate the categories [24].

## Results

### Demographics

**Fig. 1:**
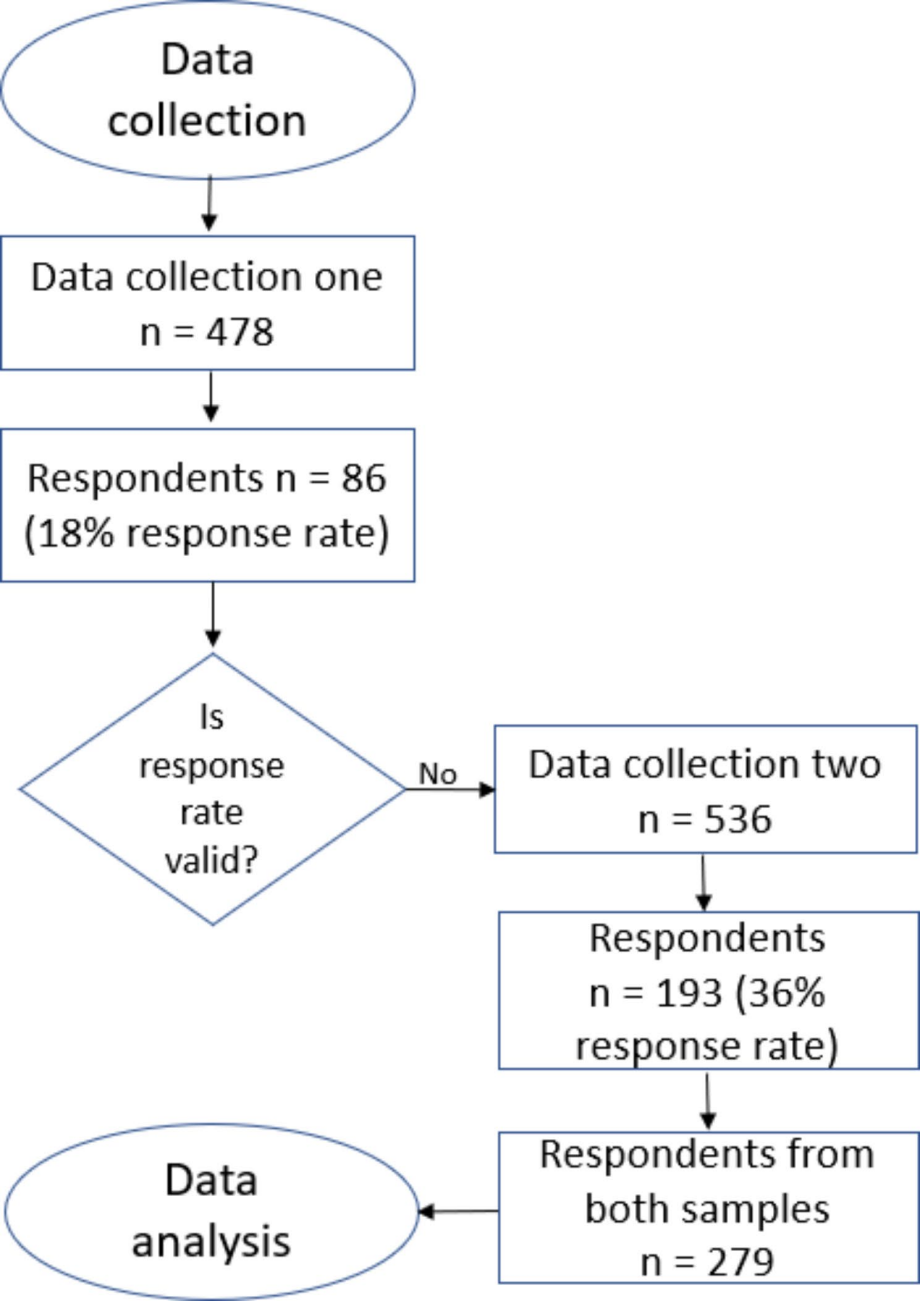
Flow chart for the data collection process

In total, 279 healthcare professionals completed the survey. In the first distribution, 478 surveys were sent out, resulting in a response rate of 18% (n = 86). The second distribution, through social media, resulted in 193 completed responses (out of 536 hits on the link to the survey), equivalent to a response rate of 36%. Respondents from both distributions included healthcare professionals working in different specialist settings, as well as primary and secondary healthcare (Fig. 1).

In sample one 26% (n = 22) were physicians compared to 13% (n = 25) in sample two. Respondents in sample one answered the free-text questions to a greater extent (90%/n = 77) in comparison to (84%/n = 162) sample two. Further, respondents in sample one reported to be more aware of the concept of patient lead users (“spetspatienter” in Swedish) (49%/n = 42) compared to (23%/n = 44) in sample two. No other differences between the samples were found. The demographic characteristics of both samples are presented in Table 1. They show a fair distribution across age, gender, workplace, and occupation. However, specialist healthcare professionals are somewhat over-represented, as well as women, reflecting the female dominance in Swedish health care.

**Table 1:** Characteristics of respondents from the first and second data collection

Age n (%) 1st data collection 2nd data collection		Gender n (%) 1st data collection 2nd data collection			Workplace (multiple answers) n (%) 1st data collection 2nd data collection		Occupation n (%) 1st data collection 2nd data collection		
**18–29**	9 (10) 21(11)	**Men**		19 (22) 8 (4)	**Primary healthcare**	0 (0) 70 (37)	**Nurse**		19 (22) 110 (57)
**30–39**	16 (19) 56 (29)	**Women**		63 (73) 185 (96)	**Specialized healthcare**	86 (100) 109 (56)	**Physician**		22 (26) 25 (13)
**40–49**	18 (21) 63 (33)				**Digital healthcare**	0 (0) 3 (2)	**Other health professions with license**		35 (41) 31 (16)
**50–59**	26 (31) 42 (22)						**Non-licensed health professions**		10 (11) 27 (14)
**60-**	15 (17) 11 (5)								
**Prefer not to say**	2 (2) 0 (0)			4 (5) 0 (0)	**Other**	0 (0) 30 (16)			

The data from both data collections were summarized under three main topics, based on the nine sections from the web survey [see Additional file 1]: (1) *Patient knowledge* (including the subsections: Knowledgeable patients and informal caregivers, To learn from patients and informal caregivers, Patients and informal caregivers communicating their experiences), (2) *Innovative patient self-care behaviors* (including the subsections: Patients performing self-tracking by own initiative, Needs of alternative ways to interact with health care, Use of digital solutions to manage disease, Patient and informal caregiver innovations), and, (3) *Patients navigating the healthcare system* (including the subsections: Coordinating healthcare contact between different healthcare units, Patients’ and informal caregivers’ engagement in the healthcare unit’s development). Results are presented per topic, listing respondents’ attitudes, experiences, and perceived workplace support for each. Free-text responses are summarized under each topic, illustrated by a quote.

### Patient knowledge

The respondents showed a positive attitude towards knowledgeable patients (96%/n = 268) and informal caregivers (97%/n = 270) and stated the importance of and their interest in effective knowledge exchange. The respondents were also positive towards patients (85%/n = 237) and informal caregivers (83%/n = 231) sharing knowledge and experiences with each other, since it has the potential to lead to better everyday life experiences. However, the free-text responses revealed that individual patient knowledge might also be irrelevant if patients and informal caregivers lack an understanding of the overall situation. Further, respondents mentioned the risk of incorrect knowledge being shared, which might lead to unrealistic expectations and demands from healthcare professionals. This was described by some respondents to make it harder to build a mutual trusting patient-healthcare professional relationship.

*Intellectually, it is generally positive with knowledgeable patents and informal caregivers, however, emotionally there is one negative aspect. There is a concern of getting out of balance in my profession if they are highly competent and at the same time critical… If so, they might not feel confidence in me as a healthcare professional and this will aggravate our partnership.* (Nurse, Specialized healthcare, Data collection two)

**Fig. 2:**
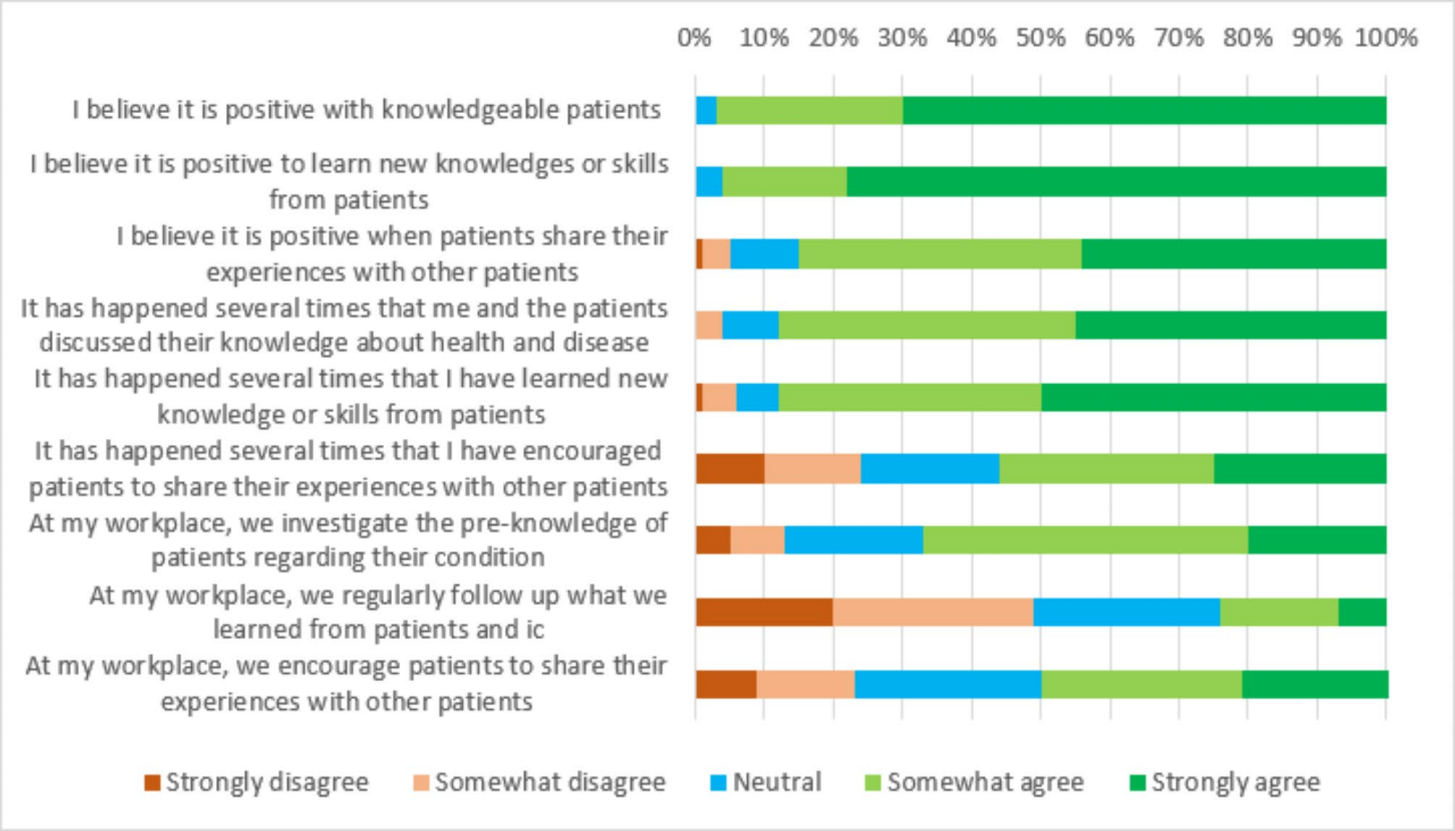
Attitudes and experiences of knowledgeable patients and informal caregivers (ic) (n = 279)

Most respondents had experiences of learning from patients (88%/n = 246) and informal caregivers (69%/n = 193). Respondents also described experiences of discussing existing knowledge with their patients (88%/n = 246) and the informal caregivers (65%/n = 181). Such discussions often included the latest standard of care, patient symptoms, and the complexity of the condition. However, few respondents stated having regular follow-ups regarding what they learned from individual patients and informal caregivers (24%/n = 67) (Fig. 2). In the free-text responses, some respondents described not having the time and support from their workplace to do so. Half of the respondents stated having experiences of encouraging patients (56%/n = 156) and informal caregivers (47%/n = 131) to share their experiences with their peers, and almost the same number of participants confirmed that their workplace supported them to do so (patients 51%/n = 142) (informal caregivers 46%/n = 128). Overall, the free-text responses showed that respondents had great support from their co-workers regarding existing challenges with demanding patients and informal caregivers, but less support from existing structures within their workplace and from operational management.

### Innovative patient self-care behaviors

**Fig. 3:**
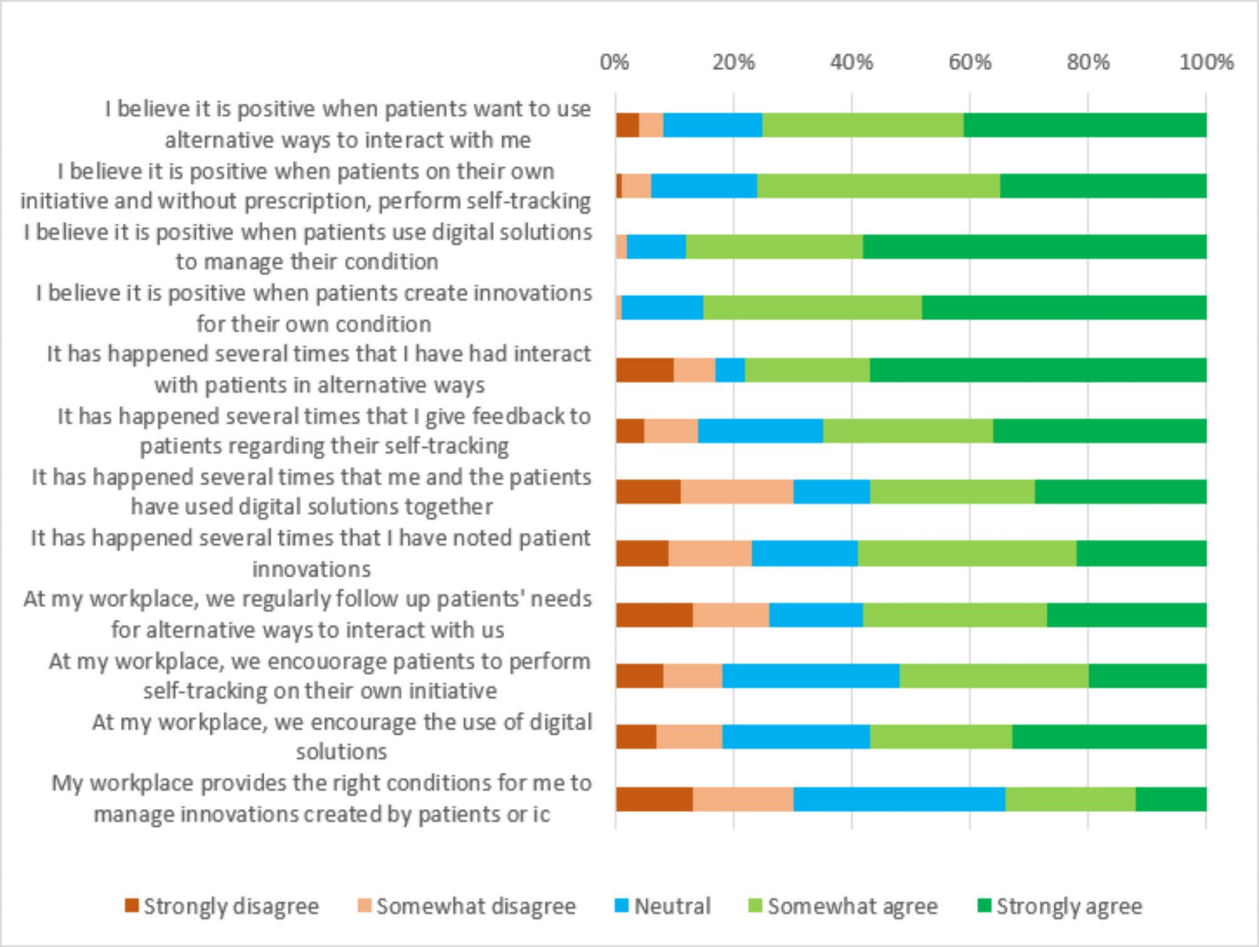
Attitudes and experiences of innovative patient self-care behaviors (n = 279)

Most participants reported a positive attitude towards patients (85%/n = 237) and informal caregivers (76%/n = 212) creating innovations for their own health conditions. Similar results were reported for patients (88%/n = 245) and informal caregivers (77%/n = 215) that used digital solutions to manage their own condition. The respondents also showed a positive attitude towards patients (75%/n = 209) that use alternative ways to interact with health care. Patients who perform self-tracking on their own initiative were considered positive (76%/n = 212) by the participants. A majority of the respondents confirmed having given feedback various times to patients own collected data (65%/n = 181) (Fig. 3). More than half of the respondents had experiences of using digital solutions together with patients (57%/n = 159) to some degree, however it was less common together with informal caregivers (33%/n = 92). The respondents also reported some experience from patient innovations (59%/n = 165), but less from innovations performed by informal caregivers (44%/n = 123). However, only 34% (n = 95) of the respondents perceived that their workplace provided the right conditions for them to manage innovations created by patients or informal caregivers. Just over half of the respondents (58%/n = 162) stated to have a workplace that regularly followed up patients’ needs for alternative ways to interact. Half of the respondents considered their workplace to be encouraging towards patients performing self-tracking to some degree (52%/n = 145) (Fig. 3). Some concerns were raised related to patients using non-validated methods for self-tracking which was considered being too subjective. Even though the healthcare professionals’ attitudes were positive regarding use of digital solutions, they reported that their organizations were not encouraging to the same extent (57%/n = 159) (Fig. 3). In the free-text responses, some respondents described negative experiences such as increased anxiety for patients and informal caregivers using digital solutions, since they were not considered evidence-based. Some respondents also expressed concerns that digital solutions potentially could lead to less face-to-face interactions, increased inequality, and unnecessary additional work for healthcare professionals. Overall, the respondents reported that the main challenges were lack of time and flexibility when engaging with empowered patients and informal caregivers.

*To ”google” your symptoms and condition might be very misleading. Since treatments can be individually adapted, these are often questioned (mainly from informal caregivers) which leads to long discussions that we do not have time for, unfortunately.* (Nurse, Specialized healthcare, Data collection two)

### Patients and informal caregivers navigating the healthcare system

**Fig. 4:**
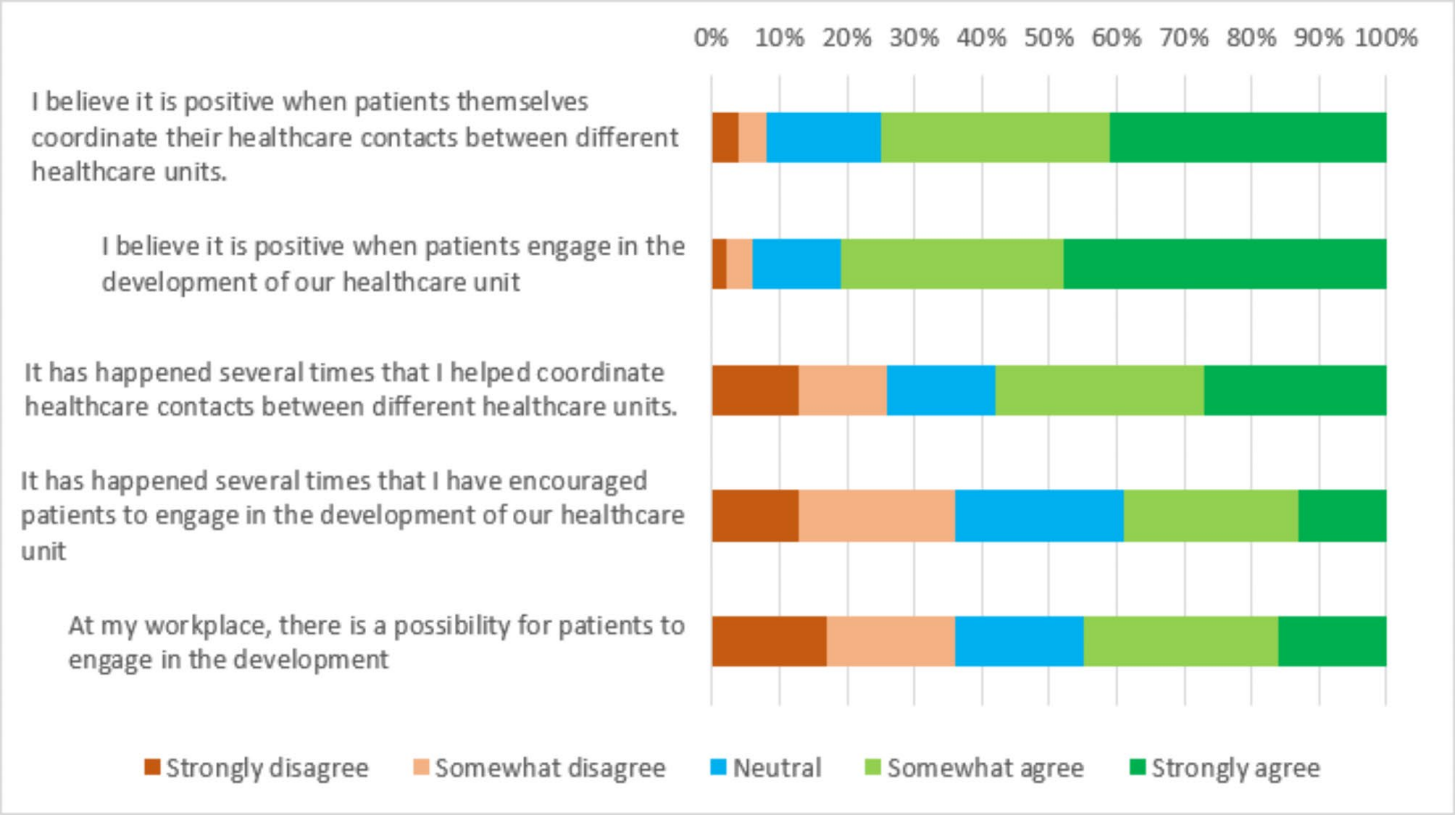
Attitudes and experiences of patients navigating the healthcare system (n = 279)

Most respondents had positive attitudes towards patients engaging in the development of health care (81%/n = 226) (Fig. 4). It was not considered equally positive when informal caregivers engaged in the development (66%/n = 184). A majority of the respondents showed positive attitudes towards patients (75%/n = 209) and informal caregivers (78%/n = 218) coordinating their own healthcare contacts between different healthcare units. Some respondents raised a concern that not everyone can or should coordinate their own health care. The free-text responses also indicated that the respondents considered themselves helping patients and informal caregivers coordinating their healthcare contacts. However, only 58% (n = 162) confirmed to be doing so in the closed survey questions (Fig. 4). Even though it was considered positive with patients and informal caregivers navigating the healthcare system in different ways, relatively few respondents stated that they had experience of encouraging patients (39%/n = 109) to engage in the development of the clinical workplace. Even fewer respondents reported having encouraged informal caregivers to do so (27%/n = 75). Respondents’ experiences were often exclusively connected to engagement with patients, but less with informal caregivers (as this group was not considered always representing the patients’ needs, and as a group considered more difficult to satisfy). However, in the free-text responses some of the respondents reported trying to involve informal caregivers in the patients’ care. Some respondents saw possibilities for patients (45%/n = 126) and informal caregivers (33%/n = 92) to engage in the development of the clinical workplace, because of existing inadequacies in the healthcare system. These inadequacies could be lack of organizational guidelines, routines, resources, and forums for engagement.

*It is difficult to encourage this when there is no existing plan for it within our organization.* (Physician, Specialized healthcare, Data collection two)

## Discussion

Overall, the healthcare professionals participating in this survey revealed a positive attitude towards empowered patients and informal caregivers, despite lack of experiences of working together with them. Only a small proportion of respondents considered their organizations to provide the optimal conditions to involve patients and informal caregivers as well as support the respondents when difficult situations occurred. The questionnaire developed for this study was based on the self-empowering behaviors found by Scott Duncan et al. [11]. Meeting the influencing self-empowering behaviors [11] showed that our respondents had positive attitudes towards patients (85%) and informal caregivers (83%) sharing their knowledge with other peers as mentors or communicators. The respondents had encouraged this to some degree, and it existed some organizational support. It was as well positive attitudes regarding patients (85%) innovating for their needs, as well as for informal caregivers (76%). The respondents were to some degree involved in patient and informal caregiver innovations. However, the support from their organization regarding managing these innovations was rather low (34%). Being engaged in healthcare development as activist or healthcare partner was considered positive if being a patient (81%) and somewhat positive being an informal caregiver (66%). However, workplace support was rather low for involving patients (45%) and informal caregivers (33%) in healthcare development. Even though there are overall positive attitudes from healthcare professionals, it was reported in Scott Duncan et al. [11] that patients and informal caregivers considered having low support for their efforts and wished to do more than was expected from them by healthcare professionals [11]. The respondents also considered it to be positive when patients (75%) and informal caregivers (78%) coordinated their own health and care, whereas patients and informal caregivers considered coordinating their own care as a burden [11]. The respondents did as well have less experience of helping out coordinating the care (58%), even though patients and informal caregivers have reported a need for better support [11].

The participants’ positive attitudes are contradictory to other studies [25, 26], where rather negative attitudes are displayed from healthcare professionals regarding patient involvement. Less experiences of working together with empowered patients and informal caregivers among our respondents could be the result of different barriers for patient involvement. One barrier reported in the literature is lack of communication and confidence of physicians [27, 28]. Other barriers reported are the paternalistic structure within the healthcare organization and lack of time and encouragement [28, 29]. It seemed to be even more difficult to involve informal caregivers for our respondents. The literature expresses lack of involvement, support, and being acknowledged by healthcare professionals as informal caregivers [30–32] as a confirmation of these barriers.

A weakness of our study is the low response rate. Still, the sample included a total of 279 participants in an online survey despite a demanding time in society and health care because of the COVID-19 pandemic. Web surveys also tend to generate lower average (44,1%) in response rate than paper and telephone surveys [33]. Not using a validated survey could be a weakness, however, both cognitive interviews and pilots were performed to ensure the relevance of the questions. A non-probability sampling was used, which might have caused a selection bias and a skewed sample [23], since we do not know if the respondents were more positive towards empowered patients and informal caregivers in general. Given the healthcare professionals’ positive attitudes, future research needs to investigate how healthcare systems can better meet the willingness to involve empowered patients and informal caregivers to a larger extent.

## Conclusion

The healthcare professionals who responded to this survey lacked experiences of working with empowered patients and informal caregivers, and workplace support for involving patients and informal caregivers. It was considered difficult to engage empowered patients and informal caregivers in the way the respondents preferred, and could lead to less active patient participation. The overall positive attitudes of healthcare professionals suggest however possible implications for health care and policymakers to provide better structures for involving patients and informal caregivers. These positive attitudes are a fundamental prerequisite towards a transition of the healthcare system into recognizing empowered patients and informal caregivers as partners.

## Electronic supplementary material

Below is the link to the electronic supplementary material.

Supplementary Material 1

## Data Availability

According to the EU General Data Protection Act personal data is restricted and anonymized. Data in Swedish is available by the corresponding author.
