# Supplementary material for: Empowered patients and informal care-givers as partners?—a survey study of healthcare professionals’ perceptions
Source: BMC Health Serv Res. 2023 Apr 26;23:404. doi: 10.1186/s12913-023-09386-8 (PMC10131407; doi:10.1186/s12913-023-09386-8)
Supplement: Supplementary file 1 — Supplementary Material 1 [file 12913_2023_9386_MOESM1_ESM.docx]

## Demographics

**Age:** 19-29 years, 30-39 years, 40-49 years, 50-59 years, 60-69 years, >70 years
**Gender:** Woman, Man, Other option, Prefer not to say
**Region:** Free text option *
**Workplace:** Primary care, Specialized health care, Digital care, Other *
**Healthcare unit:** Free text option *
**Occupation:** Free text option *
**For how long have you been working in health care?** <3 months, 3-12 months, >12 months *
**Are you aware of the concept “e-patients”?** Yes, No *
**Comment:** Free text option

## Knowledgeable patients and informal caregivers

All statements have the same answering possibilities: Strongly agree, Agree somewhat, Neither agree nor disagree, Disagree somewhat, Strongly disagree.

**I believe it is positive with knowledgeable patients. ***

**At my workplace, we investigate the pre-knowledge of patients regarding their condition**. *

**It has happened several times that me and the patients discussed their knowledge about health and disease. ***

**I believe it is positive with knowledgeable informal caregivers. ***

**At my workplace, we investigate the pre-knowledge of informal caregivers regarding the patient’s condition**. *

**It has happened several times that me and the informal caregiver discussed their knowledge about health and disease. ***

**Comment:** Free text option

## To learn from patients and informal caregivers

All statements have the same answering possibilities: Strongly agree, Agree somewhat, Neither agree nor disagree, Disagree somewhat, Strongly disagree.

**I believe it is positive to learn new knowledge or skills from patients. ***

**It has happened several times that I have learned new knowledge or skills from patients. ***

**I believe it is positive to learn new knowledge or skills from informal caregivers. ***

**It has happened several times that I have learned new knowledge or skills from informal caregivers. ***

**At my workplace, we regularly follow up what we have learned from patients or informal caregivers. ***

**Comment:** Free text option

## Need for alternative ways to interact with health care

All statements have the same answering possibilities: Strongly agree, Agree somewhat, Neither agree nor disagree, Disagree somewhat, Strongly disagree.

**I believe it is positive when patients want to use alternative ways to interact with me, than through physical encounters. ***

**At my workplace, we regularly follow up patients’ needs for alternative ways to interact with us. ***

**It has happened several times that I have had interact with patients in alternative ways, than through physical encounters. ***

**Comment:** Free text option

## Coordinating healthcare contacts between different healthcare units

All statements have the same answering possibilities: Strongly agree, Agree somewhat, Neither agree nor disagree, Disagree somewhat, Strongly disagree.

**I believe it is positive when patients themselves coordinate their healthcare contacts between different healthcare units. ***

**I believe it is positive when informal caregivers coordinate the patient’s healthcare contacts between different healthcare units. ***

**It has happened several times that I helped coordinate healthcare contacts between different healthcare units. ***

**Comment:** Free text option

## Patients performing self-tracking on their own initiative

All statements have the same answering possibilities: Strongly agree, Agree somewhat, Neither agree nor disagree, Disagree somewhat, Strongly disagree.

**I believe it is positive when patients on their own initiative and without prescription, perform self-tracking of e.g. symptoms. ***

**It has happened several times that I give feedback to patients regarding the self-tracking they performed on their own initiative. ***

**At my workplace, we encourage patients to perform self-tracking on their own initiative. ***

**Comment:** Free text option

## Use of digital solutions (e.g. 1177, diagnosis specific applications) to manage health conditions

All statements have the same answering possibilities: Strongly agree, Agree somewhat, Neither agree nor disagree, Disagree somewhat, Strongly disagree.

**I believe it is positive when patients use digital solutions to manage their condition. ***

**It has happened several times that me and the patients have used digital solutions together. ***

**I believe it is positive when informal caregivers use digital solutions to manage the patient’s condition. ***

**It has happened several times that me and the informal caregivers have used digital solutions together. ***

**At my workplace, we encourage the use of digital solutions. ***

**What negative aspects do you perceive when digital solutions to manage health conditions are used?** Free text option

**Comment:** Free text option

## Innovations by patients and informal caregivers

All statements have the same answering possibilities: Strongly agree, Agree somewhat, Neither agree nor disagree, Disagree somewhat, Strongly disagree.

**I believe it is positive when patients create innovations for their own condition (innovations = new for the patient group, simplify everyday life, contribute with increased, perceived health). ***

**It has happened several times that I have noted patient innovations. ***

**I believe it is positive when informal caregivers create innovations for the patient’s condition. ***

**It has happened several times that I have noted innovations performed by informal caregivers. ***

**My workplace provides the right conditions for me to manage innovations created by patients or informal caregivers. ***

**Comment:** Free text option

## Patient and informal caregivers communicating their experiences

**I believe it is positive when patients share their experience with other patients. ***

**At my workplace, we encourage patients to share their experiences with other patients**. *

**It has happened several times that I have encouraged patients to share their experiences with other patients. ***

**I believe it is positive when informal caregivers share their experience with other informal caregivers. ***

**At my workplace, we encourage informal caregivers to share their experiences with other informal caregivers**. *

**It has happened several times that I have encouraged informal caregivers to share their experiences with other informal caregivers. ***

**Comment:** Free text option

## Patients’ and informal caregivers’ engagement in the healthcare unit’s development

**I believe it is positive when patients engage in the development of our healthcare unit. ***

**At my workplace, there is a possibility for patients to engage in the development.** *

**It has happened several times that I have encouraged patients to engage in the development of our healthcare unit. ***

**I believe it is positive when informal caregivers engage in the development of our healthcare unit. ***

**At my workplace, there is a possibility for informal caregivers to engage in the development.** *

**It has happened several times that I have encouraged informal caregivers to engage in the development of our healthcare unit. ***

**Comment:** Free text option

## Free text options

**What do you consider is the best with your workplace regarding how you collaborate with patients and informal caregivers?**

**What challenges do you perceive when patients and informal caregivers wish to be more engaged?**

**What support exists within your workplace for existing challenges?**
